# Viridiplantae Body Plans Viewed Through the Lens of the Fossil Record and Molecular Biology

**DOI:** 10.1093/icb/icac150

**Published:** 2022-10-31

**Authors:** Karl J Niklas, Bruce H Tiffney

**Affiliations:** The School of Integrative Plant Science, Cornell University, Ithaca, NY 14853, USA; Department of Earth Science and College of Creative Studies, University of California, Santa Barbara, CA 93106, USA

## Abstract

A review of the fossil record coupled with insights gained from molecular and developmental biology reveal a series of body plan transformations that gave rise to the first land plants. Across diverse algal clades, including the green algae and their descendants, the plant body plan underwent a unicellular $\to $ colonial $\to $ simple multicellular → complex multicellular transformation series. The colonization of land involved increasing body size and associated cell specialization, including cells capable of hydraulic transport. The evolution of the life-cycle that characterizes all known land plant species involved a divergence in body plan phenotypes between the haploid and diploid generations, one adapted to facilitate sexual reproduction (a free-water dependent gametophyte) and another adapted to the dissemination of spores (a more water-independent sporophyte). The amplification of this phenotypic divergence, combined with indeterminate growth in body size, resulted in a desiccation-adapted branched sporophyte with a cuticularized epidermis, stomates, and vascular tissues. Throughout the evolution of the land plants, the body plans of the sporophyte generation involved “axiation,” i.e., the acquisition of a cylindrical geometry and subsequent organographic specializations.

“A fixed cylindrical body has the potential to resist gravity by having a design with a high flexural stiffness while retaining the ability to reach out.”

–– Stephen A. Wainwright, Axis and Circumference (Wainwright SA. 1988, p. 95)

## Introduction

If it is true that the present is a key to understanding the past (Gitzendanner et al. 2018), it is equally true that the past is a key to understanding the present (Rothwell et al. 2018). The conceptual reciprocity obtained by studying organisms from the perspective of molecular biology and the perspective of the fossil record is critical to understanding evolution (e.g., Wilson et al. 2017). The goal of this paper is to apply this strategy to review the evolution of plant body plans using information drawn from the fossil record and molecular biology. Plants are broadly defined here to include any photosynthetic eukaryote, thereby including the polyphyletic algae (Graham et al. 2009). However, we focus primarily on the monophyletic land plants (i.e., the embryophytes) and their antecedents, the monophyletic green algae (i.e., Chlorophyta), thereby providing sufficiently broad comparisons.

**Fig. 1:**
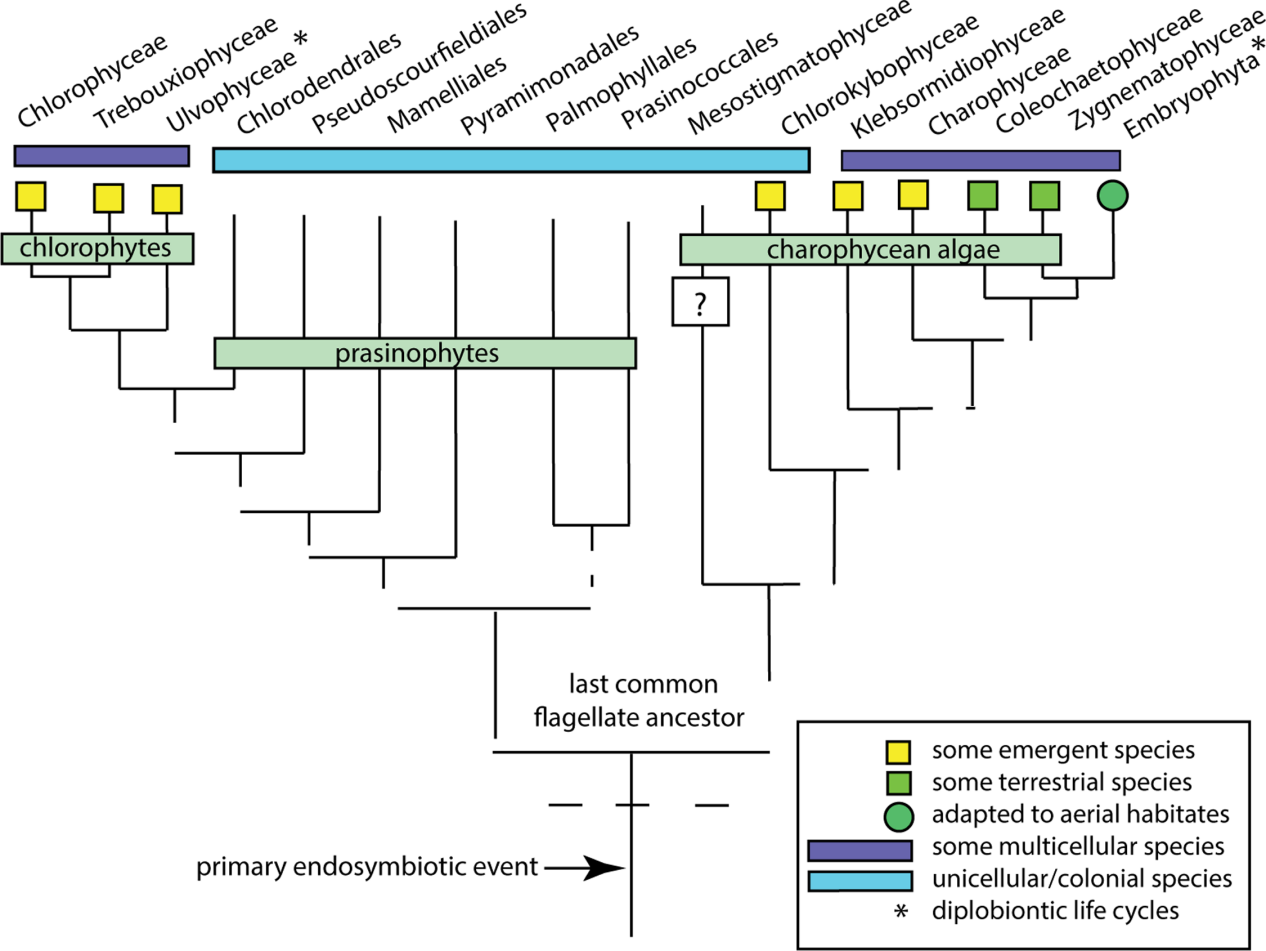
Redacted phylogenetic tree showing the relationships among the lineages within the Viridiplantae. This schematic also diagrams some of the shared features between the major lineages within the clade (i.e., the chlorophytes, prasinophytes, and streptophytes). The latter include the charophytes and the embryophytes, embracing the evolution of a diplobiontic life cycle (see insert at lower right). Some of the relationships are problematic, as indicated by broken lines. For example, some molecular analyses place the Zygnematophyceae closer to the land plants (the embryophytes) than either the Charophyceae or the Coleochaetophyceae (adapted from Lewis and McCourt, 2004).

Our goal is motivated by the fact that little has been written about the evolution of plant body plans, and the very little that has been written has focused largely on the vascular land plants (e.g., Banks, 1975; Stewart and Rothwell, 1993; Taylor et al. 2009; Kaplan, 2022; see, however, Niklas, 2000) to the neglect of what came before the appearance of vascular plants. This inattention is particularly striking because molecular phylogenetic analyses clearly indicate that the embryophytes are the direct descendants of one lineage within the Chlorophyta, justifying the inclusion of the green algae and the land plants in a monophyletic clade, the Viridiplantae (Liu et al. 2014; Wickett et al. 2014; Cox, 2018; Puttick et al. 2018). In addition, molecular phylogenetic analyses of the Chlorophyta indicate that the unicellular body plan is the ancestral condition, as is likely for all very ancient lineages, and that multicellularity is a highly derived condition (Fig. 1). Consequently, any attempt to reconstruct the evolution of plant body plans requires a broad assessment of how multicellularity and the diverse traits characterizing the Viridiplantae emerged from something as seemingly simple as a unicellular photosynthetic cell.

The following sections (1) present the developmental processes required for a unicellular-to-multicellular transition, (2) consider a scenario for the transition from simple to complex multicellularity, and (3) review the evolution of the land plants based on the fossil record and molecular data in light of the information arising from (1) and (2). One emerging theme is the evolution of “axiation,” i.e., the emergence of a cylindrical geometry as an optimal solution to the competing functional obligations that multicellular photosynthetic organisms must perform. A second theme is the phenotypic convergence on axiation seen between the two multicellular generations in the land plant life cycle, i.e., the haploid gametophyte and the diploid sporophyte (Kenrick, 2017). A main thesis is that axiation was a central adaptation, starting with the first elongate cells and culminating in some of the largest multicellular autotrophs such as kelps and sequoias.

## Developmental motifs

Setting aside the similarities among unicellular or colonial species, which are arguably trivial owing to their simple morphologies, extensive phenotypic diversification and convergence are evident within the Viridiplantae. Consequently, it is often impossible to distinguish between species drawn from different lineages within the same clade based on their general appearance, size, or internal structure (Bold, 1967; Bierhorst, 1971; Bold and Wynne, 1978; Graham and Wilcox, 2000; Graham et al. 2009). Indeed, the degree to which internal structure (i.e., anatomy) has diverged in some lineages is so great that some workers claim that anatomy cannot be used to understand morphological homologies (Kaplan, 2022). The perspective taken here is that body plans are more profitably discussed in terms of how they are achieved developmentally rather than discussing them in terms of their resulting phenotypes, given the extensive morphological and anatomical convergence and divergence among the various plant lineages (e.g., Graham et al. 2009; LoDuca et al. 2017; Thorogood et al. 2017).

**Fig. 2:**
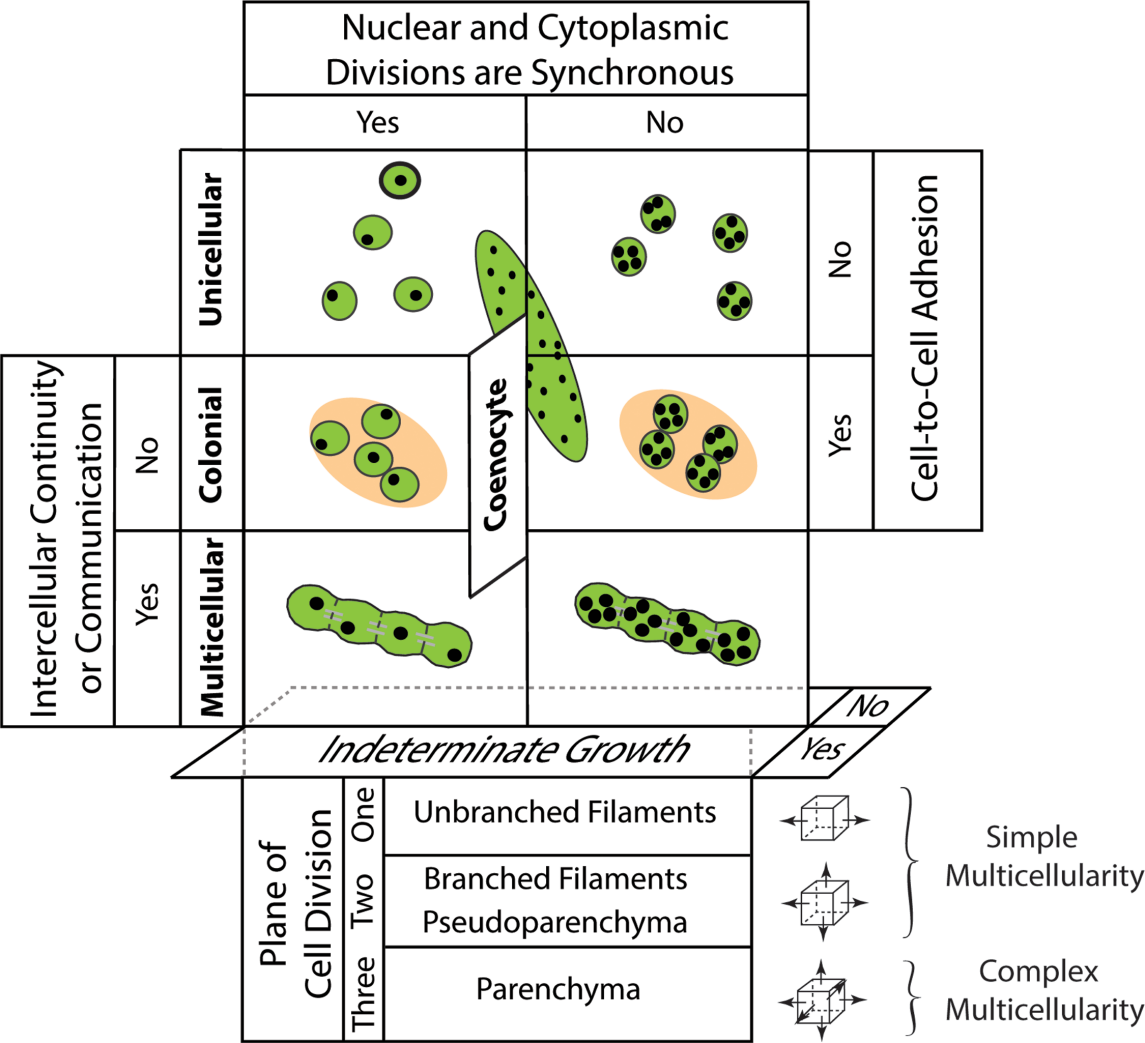
A schematic of the developmental motifs that result in four plant body plans (i.e., the unicellular, colonial, coenocyte, and multicellular body plans). The unicellular body plan is achieved by the separation of cell division products after cytokinesis. The colonial body plan is a collection of uni- or multinucleate cells aggregated by extracellular adhesives but lacking intercellular continuity among cells. The unicellular and colonial body plans are determinate in cell size, although the overall size of a colony may increase by the addition of cells. The coenocyte body plan is indeterminate in its growth in size (e.g., *Caulerpa*). The multicellular body plan consists of uni- or multinucleate cells that maintain intercellular continuity after cytokinesis (e.g., the unbranched filamentous chlorophytes *Ulothrix* and *Urospora*, respectively) (for additional clarification, see Fig. 3).

This approach identifies four body plans distinguished on the basis of a few simple developmental motifs: the unicellular, colonial, coenocytic, and multicellular body plans (Fig. 2). Each body plan requires the interaction of four processes (Niklas, 2000): (1) the presence or absence of synchronous cyto- and karyokinesis, which determines whether the body plan consists of uni- or multinucleate cells (e.g., *Chlamydomonas* and *Bryopsis*, respectively); (2) the separation of derivative cells or their aggregation by means of an extracellular adhesive, which determines whether the body plan is unicellular or colonial (e.g., *Chlorella* and *Hydrodictyon*, respectively); (3) whether individual cells manifest indeterminate growth resulting in a coenocytic body plan or not (e.g., *Caulerpa* and *Chlorella*, respectively); and (4) whether intercellular continuity persists during and after cell division by means of cytoplasmic bridges, plasmodesmata, etc., which distinguishes the colonial from the multicellular body plan (e.g., *Scenedesmus* and *Chara*, respectively). The multicellular body plan in this scheme has three variants based on the planes of cell division with respect to the body axis (Fig. 2): (1) Unbranched filaments result when the plane of division is confined to one plane of orientation with respect to the body axis (e.g., the green alga *Spirogyra*); (2) branched filaments and monostromatic cell constructs can form when the plane of division is confined to two planes of cell division (e.g., *Stigeoclonium* and *Volvox*, respectively); the former can be used to construct pseudoparenchymatous tissues (e.g., *Codium*); and (3) a complex multicellular body plan is achieved when cell division occurs in all three planes (e.g., all land plants).

The scheme shown in Fig. 2 does not drive a conceptual wedge between the different generations in the life cycles of individual species; e.g., some green algae possess the same body plan in both the haploid and diploid generations (e.g., *Ulva*). However, land plants have a dimorphic life cycle in which haploid and diploid generations differ in size, morphology, or anatomy. Consequently, Fig. 2 can be modified to include the two ploidy levels in the land plant life cycle and their respective abilities to produce secondary tissues such as bark and secondary xylem.

The scheme shown in Fig. 2 reveals the extent to which body plans have both diversified and converged within and among different clades. For example, unicellular, colonial, coenocyte, and multicellular body plans occur in the Chlorophyta and the golden-brown algae, the Chrysophyta. Likewise, the three variants of the multicellular body plan (i.e., unbranched filaments, branched filaments, and complex multicellular bodies) occur in the red and brown algae, as well as in distantly related lineages within the Viridiplantae (see Fig. 1). However, unlike the charophycean algae, which have unicellular and colonial representative species (e.g., *Stichococcus* and *Chlorokybus*, respectively), and multicellular species (e.g., *Chara* and *Coleochaete*), all land plants are multicellular and constructed out of parenchymatous tissue systems, although all variants of the multicellular body plan are expressed at different times during development (e.g., the unbranched and branched filaments of moss protonema as well as in the complex bodies of sporophytes such as trichomes and pseudoelators). Likewise, the coenocytic body plan is expressed transiently among some land plants (e.g., the “free-cellular” condition of endosperm and megagametophytes; see Bierhorst, 1971).

## The evolution of multicellularity

The ability of cells to adhere is necessary but not sufficient to achieve multicellularity. Adhesion occurs among all unicellular and colonial species, whereas the control of the planes of cell division is required to achieve an organized multicellular body plan. Multicellularity requires a systemic spatial reference system (SSRS). This appears to be ancient among prokaryotes. Precambrian filamentous prokaryotes exhibit simple multicellularity (Schopf and Barghoorn, 1967; Knoll, 2015). Complex multicellularity appears to have evolved only within the eukaryotes.

**Fig. 3:**
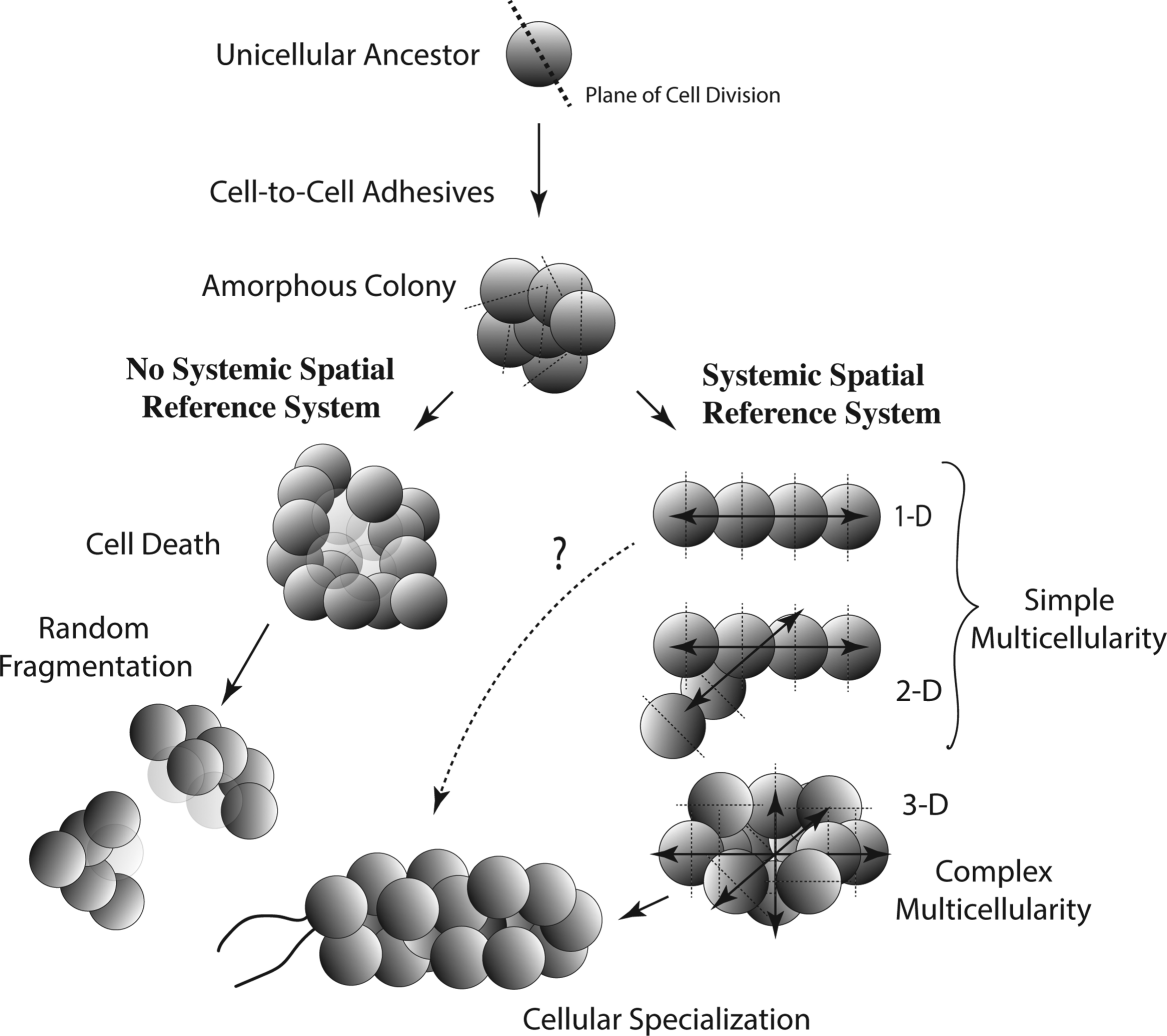
Schematic of how simple and complex multicellularity may have evolved. The proximal condition is assumed to have had a colonial body plan, a descendant of a unicellular ancestor. In the absence of an organismic reference system, the colonial body plan lacks the capacity to organize systemic planes of cell divisions (shown to the left). By evolving an organismic reference system (possibly by means of cell-to-cell cytoplasmic connections), a colonial body plan can evolve simple (1-D and 2-D) and complex (3-D) multicellularity.

The distinction between simple and complex multicellularity may seem trivial (Fig. 3). However, when viewed through the lens of physiology, the distinction becomes important. Consider, for example, Fick’s law of passive diffusion, which shows that the time it takes for a nonelectrolyte to diffuse within a spherical cell is linearly proportional to the cell’s radius (Nobel, 2009). It increases with increasing cell volume and decreases with increasing cell surface area. The same holds true for an amorphous mass of randomly dividing cells. With continued cellular proliferation, passive diffusion becomes increasingly unable to meet the metabolic demands of cells progressively deeper within the aggregate for nutrients available only from the external environment, and conversely, for the expulsion of potentially toxic metabolites into the external environment.

There are different adaptive solutions to the metabolic and physical constraints imposed by passive diffusion. For example, aggregated cells can fragment once a colony reaches a critical size, possibly fostered by the death of internalized starved cells (e.g., palmella growth forms of *Chlamydomonas*). Cells could also achieve greater control over the orientation of their planes of division to achieve simple multicellularity (Fig. 3). Prokaryotes have gone down both pathways. Some have followed what is arguably the simpler path and produced amorphous cell masses (e.g., *Staphylococcus*). Other prokaryotes produce filaments of cells (e.g., *Anabaena*). The fragmentation of colonies can be advantageous for species living in hydrodynamically active environments. Fragmentation provides a simple method for long-distance dispersal and colonization. Simple multicellularity provides the same potential while simultaneously bypassing the constraints of passive diffusion. Cellular congeries and filaments are preserved as Precambrian microfossils estimated to be 3.8–3.4 billion years old (e.g., Schopf and Barghoorn, 1967).

A simple scenario provides a model for how complex multicellularity evolved (Fig. 3). It begins with prokaryotes producing extracellular adhesives but being incapable of systemically controlling the orientations of their planes of division. These organisms subsequently evolved and diverged in their ability to control and orient cell division. Some failed to do so, whereas others achieved the capacity to produce unbranched or branched filaments (denoted as 1–D and 2–D, respectively), thereby achieving simple multicellularity. How this integration of cell division was achieved remains problematic, although it is noteworthy that physical contact can serve as a cue between adjoining cells for aligning the plane of division. Likewise, diffusion of a metabolite between cells can create a reaction–diffusion (R–D) morphogenic system (e.g., Xu et al. 2020). Heterocyst formation in *Anabaena* and *Nostoc* filaments is an example. Heterocysts produce a diffusible inhibitor that prevents heterocyst formation unless its concentration falls below a specific threshold. As the distance between two heterocysts increases due to the division of intervening vegetative cells, the first undifferentiated cell to be triggered by the drop in the concentration gradient develops into a new heterocyst. This newly formed heterocyst releases the inhibitor and reiterates the process. Although this example deals with the morphogenesis of a specialized cell (i.e., the heterocyst), it is not difficult to imagine that the diffusion of a substance between two adjoining cells could serve as a chemical as well as a physical cue establishing the orientation of the plane of future cell division.

Interestingly, the mechanism achieving the heterocyst involves diffusion through intercellular cytoplasmic connections (sometimes called micro-plasmodesmata). These structures facilitate intercellular coordination. They also help to establish (or at the very least contribute to) body plan polarity (Niklas et al. 2019). Importantly, these intercellular connections have analogs in eukaryotic multicellular organisms, as, for example, the gap and tight junctions in metazoans, plasmodesmata in land plants, and septal pores in filamentous fungi. The convergent evolution of diverse modes of intercellular connections in disparate lineages and clades indicates the profound importance of cell-to-cell communication and coordination.

Importantly, the systemic control of planes of cell division need not reflect the immediate consequences of natural selection. Evolutionary innovations can also arise via phenotypic plasticity or from the inherent physical properties of tissues and condition-dependent developmental systems. These can subsequently become genetically assimilated into an organism’s developmental program. For example, phenotypic plasticity may help to explain why the cells within colonial life forms can achieve different metastable functionalities (reflecting different phases in the life cycle of unicellular ancestors) that presage the appearance of complex multicellularity in some lineages, e.g., colony formation in *Chlamydomonas* in response to predation or nutrient deprivation (reviewed by Niklas, 2014). Divergence in cell function among genetically identical cells contributes to fitness, particularly when some functions cannot be performed simultaneously (e.g., mitosis and motility). Likewise, the colonial body plan has advantages over the unicellular body plan, e.g., theoretically, non-defective cells can compensate for the defects of other cells or provide resources so that others can functionally specialize.

Importantly, organisms can evolve along different pathways even within closely related groups of organisms. Consider the volvocine algae, in which multicellularity likely evolved by differential modifications of cell wall layers in a unicellular *Chlamydomonas*-like progenitor (Kirk, 1998, 2005). Specifically, the walls of unicellular *Chlamydomonas* are composed primarily of hydroxyproline-rich glycoproteins and are separated into a structured outer layer and a more amorphous inner layer. Among the colonial volvocines (e.g., *Gonium*), adhering cells are interconnected by struts that preserve the features of the outer cell wall layer. Yet, among multicellular volvocine algae (e.g., *Pandorina* and *Volvox*), the entire organism is surrounded by a layer that preserves features of the ancestral outer wall layer by virtue of an unusual pattern of cell division (called palintomy), in which cells undergo multiple divisions without increasing in total cytoplasmic volume (Herron et al. 2010). High degrees of similarity in protein sequences among unicellular and multicellular volvocines support this scenario; e.g., homologs of the *Chlamydomonas* outer cell wall protein GP2 occur in *Pandorina* and *Volvox* (Adair and Appel, 1989). In contrast, cell adhesion in the evolutionarily related land plants involves a middle lamella enriched with pectins, a functionally and structurally diverse class of galacturonic acid-rich polysaccharides. This contrast in the types of Viridiplantae adhesives illustrates that natural selection works on “what’s handy” and that adaptations are opportunistic––put differently, there are many roads to Rome, but no two roads are the same.

## Axiation

Molecular phylogenetic analyses identify the charophycean algae as the sister group to the land plants (Fig. 1), although substantive debate remains as to which charophycean lineage is the immediate sister group (Delwiche and Cooper, 2015; de Vries and Archibald, 2018). The three candidates are the Charophyceae, Coleochaetophyceae, and Zygnematophyceae, all of which have an unbranched or branched filamentous body plan and a life cycle in which the only multicellular generation is haploid. Therefore, the multicellular land plant sporophyte generation is presumed to be a derived condition, as is parenchymatous tissue construction.

One scenario for the evolution of the land plant sporophyte is the co-option of the gametophyte developmental program to produce a diploid generation with comparable developmental capacities. Such a “transfer” of genetic capacity would have produced a sporophyte with the ability to undergo mitotic cell divisions before some or all of its cells underwent meiosis to produce spores and subsequent gametophytes. Delaying zygotic meiosis can confer an immediate benefit––the amplification of reproductive output––because a zygote can yield four spores following meiosis and thus only four new gametophytes. However, if a zygote delays meiosis and divides mitotically only once, each derivative cell can divide meiotically, resulting in eight, not four, haploid cells that can develop into gametophytes. Thus, delaying meiosis confers a significant reproductive advantage.

If the co-option scenario is accepted, it is reasonable to assume that the most ancient land plant life-cycle was isomorphic, i.e., the gametophyte and the sporophyte had the same general morphology. Based on extant multicellular charophycean algae, the body plan of these sporophytes would have been an unbranched or branched filament. It is noteworthy that a filament consisting of cells sharing the same general shape and size has many physiological advantages. Consider, for example, the relationship between cell surface area *S* (which affects the ability of a cell to exchange mass and energy with its external environment) and volume *V* (which serves as a proxy for the metabolic demands of a cell). Assuming the cells in a filament are cylindrical with radius *r* and length l, the surface area of a filament *S* equals 2π*r*l*n* and the volume of the filament equals π*r*^2^l*n*, where *n* is the number of cells in the filament. Thus, *S*/*V* = 2/*r*. Assuming that a uniform cell radius is maintained, a filament of cells can increase in length indefinitely without decreasing its *S*/*V*.

The co-option of the gametophyte’s developmental program to produce a sporophyte would have conferred vegetative as well as physiological advantages. Delayed zygotic meiosis *a priori* requires mitotic cell divisions within a multicellular body plan. Hypothetically, new cells could be added at a variety of locations (e.g., at the tips or sides of a filament, or by intercalary cell divisions) and still maintain a branched or unbranched filamentous body plan. However, although excellent in an aquatic environment, a filamentous body plan is incompatible with a terrestrial existence because of its high surface area with respect to volume, which makes it susceptible to dehydration. This incompatibility can be resolved by a simple-to-complex multicellular transition, which could have been achieved with the evolution of a parenchymatous tissue construction. As shown in Fig. 3, the evolution of complex multicellularity requires the “internalization” of cells. Although the *S/V* of individual cells need not be affected by this internalization, the *S/V* of an organism’s body plan can be maintained, reduced, or increased depending on the size, shape, and geometry of the organism. This is easily shown by returning to the cylinder for which *S*/*V* = 2/*r*. As noted, if *r* is held constant, *S*/*V* does not change. However, when *r* decreases or increases, *S*/*V* increases or decreases, respectively.

A multicellular cylindrical geometry is particularly adaptable to physiological and mechanical demands. As noted, its surface area to volume ratio can be adjusted. In addition, the cells located at the center of each transection are equidistant from the perimeter of each section, which gives them equal access to external resources (e.g., oxygen, carbon dioxide, water, and light). Likewise, if some centrally located cells are specialized for hydraulic conduction, they can deliver, remove, or receive materials from all other cells with equal efficiency (e.g., a central conducting strand of cells is optimal for the delivery of water and nutrients to its surrounding cells). Mechanical advantages are equally evident. Centrally located cells experience little or no tensile or compressive bending stresses induced by laterally moving water or wind relative to peripheral cells. A cylinder fixed at one end and free at the other has the potential to resist gravity by virtue of a high flexural stiffness while retaining the ability to twist or bend if laterally loaded (e.g., by a sporangium or lateral axis). Likewise, a flexible buoyant cylindrical axis can deflect and twist to resist drag (see Koehl, 2022).

All the aforementioned advantages also occur in a branched cylindrical architecture, a phenotype easily achieved if the meristems at the growing free ends of cylinders multiple. Indeed, the most ancient known land plant sporophytes are composed of branched cylindrical (sometimes tapering) axes bearing terminal or lateral sporangia, e.g., *Cooksonia* and *Baragwanathia*, respectively (Taylor et al. 2009). Nor is it surprising that an unbranched or branched cylindrical architecture is seen in the growth habits of the vast majority of extant vascular land plants (e.g., the gametophores of mosses and the stems of palms, and the stems of roses and oak trees, respectively). The principal attribute of the cylinder is its ability to establish polarity in a simple and economical way. Polarity is essential on land because gravity pulls down, wind pushes sideways, and often in a preferred direction, light generally comes from above and water and nutrients come from below. In addition, a vertical cylinder can elevate reproductive organs above ground, thereby facilitating the capture and release of gametes and propagules in the air stream.

The *de novo* evolution of a diploid sporophyte raises the issue of the effects of ploidy level on plant functional traits and morphology. Indeed, the first sporophytes can be thought of as the first autopolyploids. Polyploidy is reported to decouple variation among functional quantitative traits and is hypothesized to provide an evolutionary advantage in some lineages. For example, polyploidy can modify physiological rates (Otto and Whitton, 2000), result in cell and organ enlargement (the “gigas” effect; Stebbins, 1971; Knight and Beaulieu, 2008), cope with abiotic and biotic stress (Van de Peer et al. 2021), and help to overcome the constraints imposed by trait integration (Balao et al. 2011). In addition, polyploidy is reported to affect bryophyte mating systems and the associated evolution and maintenance of reproductive traits (Jesson et al. 2010) and has been hypothesized to provide an avenue of escape from the trap of self-fertilization inherent in monoecious pteridophytes (Klekowski and Baker, 1966). Each of these effects may have contributed to the evolution of sporophyte novelties. However, the effects of polyploidy, even among closely related species, can differ in a species-dependent manner (e.g., Soltis et al. 2010; Denaeghel et al. 2018). In addition, inferences about ploidy levels in fossil materials are problematic. The role played by polyploidy during the early evolution of the land plants therefore remains highly conjectural, albeit eminently worthy of future investigations.

## Meristems

**Fig. 4:**
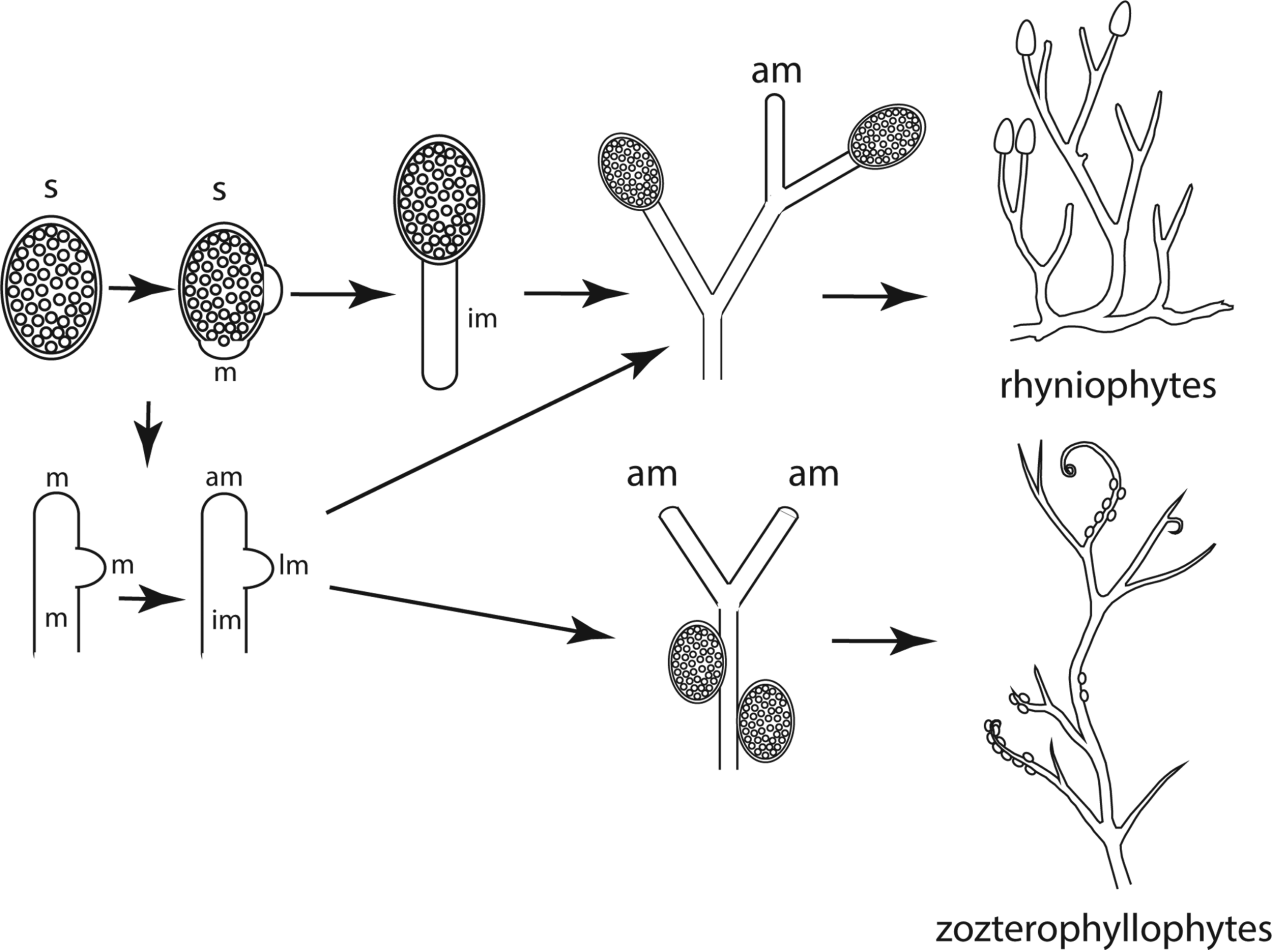
Scenarios for the evolution of the first land plant sporophyte resulting from delayed zygotic meiosis. The first multicellular land plant sporophyte could have been nothing more than a sporangium (s), i.e., a sterile “jacket” of diploid cells surrounding a mass of spores (shown in the upper left). Continued mitotic cellular divisions after the formation of spores in meristematic (m) regions would have produced additional vegetative tissues, increasing the size and elevation of the sporophyte (structures to the right). Subsequent specialization of meristematic zones into apical, lateral, and intercalary meristems (am, lm, and im, respectively; shown in the lower left) would have led to a systemic control of vegetative growth and the location of sporangia. The evolution of dedicated apical meristems that continue to produce vegetative axes would have resulted in indeterminate vegetative growth and the production of an indeterminate number of either lateral or terminal sporangia observed in the fossil record (diagrammed on the upper and lower right).

It is reasonable to argue that a cylindrical body plan provided advantages for the successful colonization of the land (or, more accurately, the air). But this body plan was not sufficient to control the size and shape of a multicellular land plant involving a cylindrical axis (e.g., bryophyte sporophytes) or a more complex construct composed of many cylindrical axes (e.g., vascular plant sporophytes), because organized multicellular growth requires a systemic spatial reference system (see Fig. 3). The mechanism seen among extant land plants is phenotypically manifest in the form of meristems, which are here defined as any cell or group of cells to which one or more lineages of derivative cells can be traced. The meristems of most extant multicellular charophycean algae consist of apical, lateral, or intercalary cells that allow unbranched and branched filamentous body plans to increase in size in an organized (often reiterative) manner. By extrapolation, the first complex multicellular land plants are likely to have had similar meristematic capabilities (Fig. 4). Indeed, the fossil sporophytes of the most ancient known vascular plant consist of simple, branched axes, some of which bear either apical or lateral sporangia. The fact that the majority of these fossils have indeterminate growth in size (as seen in the form of continued branching) indicates that meristems had become specialized to produce vegetative and reproductive body parts. Indeed, mosses and liverworts are among the very few known land plants to have sporophytes with determinate growth as a result of producing terminal sporangia on unbranched axes. The sporophytes of liverworts, mosses, and hornworts elongate by virtue of an intercalary meristem, creating the seta in the case of the former two that elevates sporangia, thereby facilitating spore dispersal.

Just as divergent genetic changes can give rise to convergent phenotypes (e.g., Povilus et al. 2020), divergent meristematic activity can give rise to the same morphologies. A review of the literature shows that the meristematic ability to produce a branched sporophyte is not the same across all vascular plant lineages. For example, among non-seed vascular plants (pteridophytes), at least three mechanisms for dichotomous branching have been reported. Bierhorst (1977) and others defined the dichotomy of an apex with a single apical cell as the establishment of two new apical cells by the division of a single apical cell, a phenomenon he observed in some filicalean ferns. Arguably, this definition is the classic conceptualization of “dichotomization” (Hofmeister, 1862; Goebel, 1928). In the second case, Roth (1963) reported that the apical cell of the aerial axes of *Psilotum* persists to become the apical cell of one of a pair of axes, while a new apical cell derived from a lineage of cells derived from the original apical cell becomes the apical meristem of the other axis. In the third mode, Gottlieb and Steeves (1961) observed that the main stem of the fern *Pteridium* ceased to grow and was replaced by the establishment of two new apical cells, a phenomenon that has also been reported for the apical meristems of the lycophyte *Selaginella* (Jernstedt et al. 1994).

A treatment of the genomic and physiological mechanisms responsible for the appearance and organization of meristems is well beyond the scope of this paper. Although the specification of the plane of cell division is a consequence, or at least a correlate, of mechanisms that rely on some form of cellular polarity, comparative analyses of diverse organisms with rigid cell walls (i.e., bacteria, algae, land plants, and fungi) indicate that the mechanisms that establish polarity (designated as POL) and the mechanisms that define the location of the future cell wall (designated as FCW) can differ even among closely related organisms (Niklas, 2000; Niklas et al. 2019), e.g., septal plugs (Brawley and Sears, 1982, and phycoplastic and phragmoplastic cell division in the Viridiplantae, Pickett-Heaps, 1976). POL can be evoked by internal cytoplasmic asymmetries and by external stimuli, e.g., gravity and unidirectional light in *Fucus* zygote development. For this reason, Hernández-Hernández et al. (2012) and Benítez et al. (2018) designated POL and FCW as plant dynamical patterning modules (DPMs), which are defined as sets of conserved gene products and molecular networks that operate in conjunction with the physical, morphogenetic, and patterning processes they mobilize (Newman and Bhat, 2009; Newman et al. 2009). Normal cell division requires that POL and FCW operate in a coordinated manner, wherein POL establishes a spatial reference system in which FCW reliably operates. Among multicellular organisms, orderly cell division typically takes place in one or more directions with respect to the body axis. Therefore, POL must establish different spatial reference systems among different plant lineages even if the mechanism responsible for POL is invariant at the cellular level. One possibility is the co-option of intercellular signaling molecules, such as auxins, particularly indole-3-acetic acid (IAA), which have been identified in charophycean algae and which are crucial for land plant development (Zažímalová et al. 2014; Zhang and van Duijn, 2014). Across the Viridiplantae, IAA mobility is driven mainly by active transport into and out of cells, but auxin influx can also result from the passive diffusion of the protonated form of IAA across the plasma membrane.

Finally, we note that the gene regulatory networks underlying the development of sporophyte axiation and branching have been the subject of intense scrutiny. However, the evolution of the genetic changes required to achieve the polysporangiate vascular plant remains an open question (e.g., Kato and Akiyama, 2005; Q. Wang et al. 2014; Y. Wang et al. 2014; Harrison, 2017). Recent reverse genetic data have implicated *PIN* and *TCP* genes in the regulation of branch initiation (Wang Q. et al. 2014, Wang Y. et al. 2014) and the suppression of axillary bud activity (Gälweiler et al. 1998; Aguilar-Martinez et al. 2007). PIN-mediated polar auxin transport is conserved between moss sporophytes and *Arabidopsis* (Fujita et al. 2008). Disruption of PIN function at low penetrance induces a branched phenotype in moss sporophytes (Harrison, 2017; Harrison and Morris, 2017). Disruption of the PpTCP5 transcription factor results in supernumerary sporangia on a single seta (Ortiz-Ramírez et al. 2016). The effects of PIN and PpTCP5 disruption on moss sporophyte development appear provocative until one realizes that they shed little direct light on the gene regulatory networks controlling cell competency and differentiation and meristematic responses. For example, auxin is a signaling molecule with manifold effects across all land plant lineages and the green algae (Cook et al. 2002), and its disruption likely has systemic effects. Until such time that the gene regulatory networks underlying shoot development are dissected more completely, it is premature to speculate about the genetic basis for sporophyte axiation and branching, particularly early in the history of the land plants.

## Growing in the air––chemical diversity and cell specialization

Although the last common ancestor to the land plants may have been aquatic or semi-aquatic, the first multicellular plants exposed to the air experienced a number of challenges, such as coping with the biomechanical stresses produced by gravity, significant temperature fluctuations, higher light intensities (particularly in the UV range), and potentially lethal water loss to the atmosphere. These challenges required metabolic adaptations from the ancestral condition, including the acquisition of structurally rigid cell walls, UV protection, and hydrophobic extracellular polymers that could impede the rapid loss of water molecules. Among extant land plants, these functional tasks are achieved primarily by four hydrophobic biopolymers (sporopollenin, lignin, cutin, and suberin), all four of which are produced by a very few metabolic pathways, some of which serve as the foundation to other critical biosynthetic pathways, as illustrated by the shikimate pathway that provides the precursors to auxin, glucosinolates, tannins, suberin, lignin, and many other compounds (Haslam, 1993; Hermann and Weaver, 1999).

Importantly, the shikimate pathway is highly conserved across bacteria, fungi, algae, and land plants. For example, homologs of the phenylpropanoid biosynthetic pathway genes have been identified in the moss *Physcomitrium patens* (formerly *Physcomitrella patens*) (Ye and Zhong, 2022), whereas the *Arabidopsis* orthologues controlling the first enzymatic step in the shikimate pathway are found in *Chlamydomonas* and *Volvox* (Tohge et al. 2013). Similarly, cytochrome P450, which catalyzes the first irreversible step committed to the biosynthesis of monolignols in angiosperms, is involved with the synthesis of phenolic components in the cuticular membrane of *Physcomitrium patens* (Renault et al. 2017). Further, both the capacity to produce sporopollenin, which protects spores and pollen from mechanical damage and desiccation, and the biosynthetic ability to produce lignin-like molecular moieties occur in green algae. For example, the cell walls of the zygote wall and placental-like transfer cells of the green alga *Coleochaete* contain sporopollenin and material similar to lignin, respectively (Delwiche et al. 1989). These and other examples of polymers similar or identical to sporopollenin, lignin, cutin, and suberin in the green algae testify to the comparative ease with which ancient biosynthetic pathways could have been co-opted to produce polymers that structurally reinforce and chemically protect cell walls and tissues, thereby permitting the first land plants to live and reproduce in the air.

**Fig. 5:**
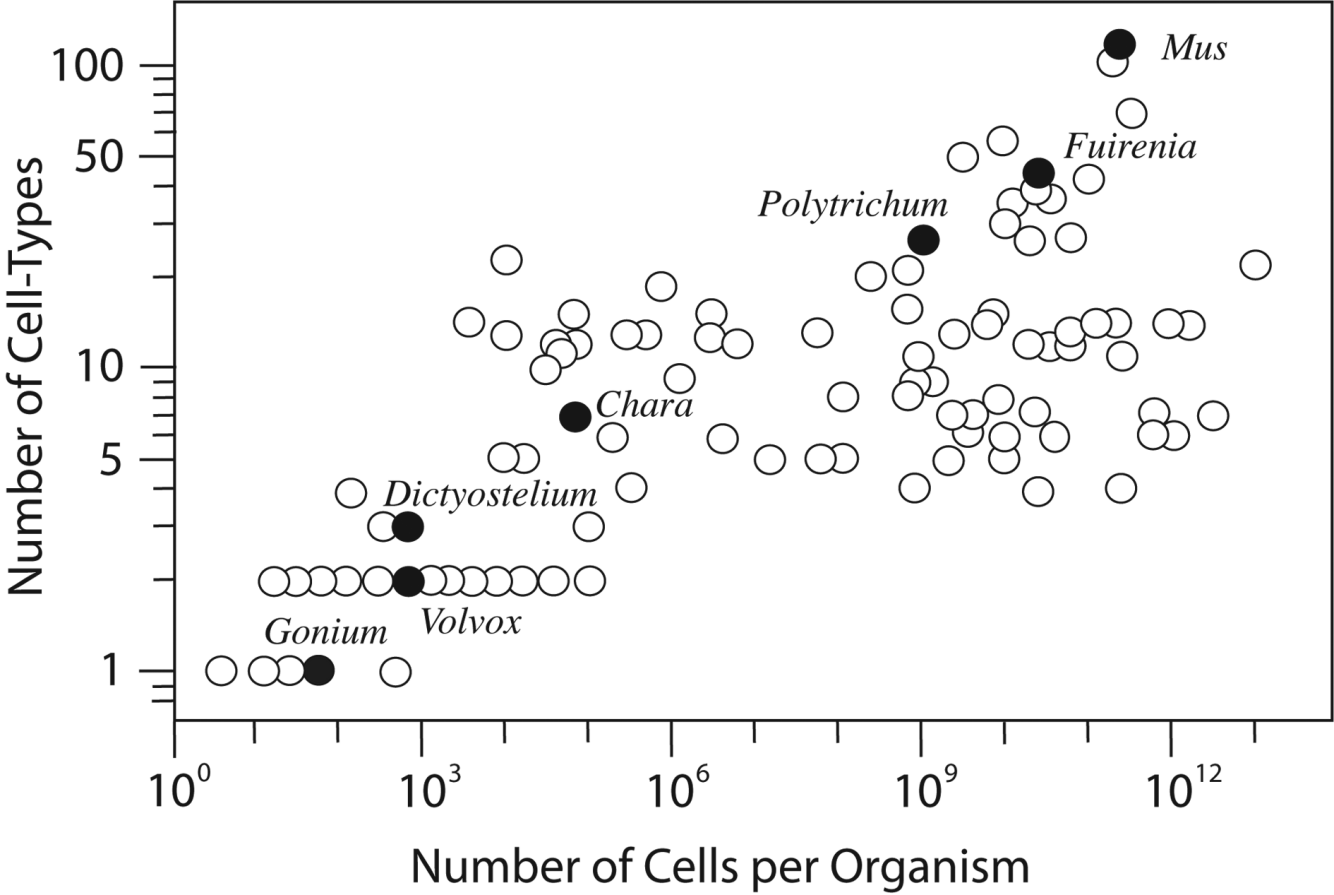
A bivariate plot of the number of specialized cell-types identified in plants, animals, and fungi versus organism cell number as gauged by the number of cells per organism. Green dots: *Gonium, Volvox*, and *Chara* are green algae; *Polytrichum* is a moss; *Fuirena* is an angiosperm; and *Dictyostelium* is a “slime mold.” Data are taken from Bell and Mooers, 1997.

This pre-existing biosynthetic versatility was paired with an increasingly specialized range of cell-types that evolved concomitantly with the diversification of indeterminate plant growth forms. Specifically, as terrestrial plants grew larger, passive diffusion could no longer accommodate the nutrient and water demands of living tissue, and new cell types and associated polymers were needed to address mass transport of fluids, the effects of desiccation, and the pull of gravity. Indeed, up to a limit, a statistically robust correlation between body size and the number of specialized cell-types across diverse animal and plant species has long been observed (Fig. 5). Although involving a wide range of cell-types, we focus here on one subset, the hydraulically specialized cells in the land plants, including the hydroids and leptoids seen in the sporophytic axes (i.e., gametophores) of many moss species (Hébant, 1977) and their functional analogues, xylem and phloem, seen in vascular plant sporophytes. It is often not recognized that cell walls lacking lignin are essentially “invisible” to the movement of water molecules by virtue of the comparatively large spaces among the hydrocarbon long-chain polymers in the primary cell wall (Wayne, 2018). Therefore, for very small terrestrial plants (e.g., <15 cm in height), particularly those living in humid microenvironments, hydraulically specialized cells are not necessary; water can simply move throughout the plant body by diffusion. Indeed, the water within hydroids can be thought of as an internal reservoir that can be drawn upon to stave off dehydration. However, for taller plants, the rate at which water passes from one cell into another is limiting, particularly when water must pass through cytoplasm. Hydroids and xylem cell-types are dead cells lacking cytoplasm. Because the storage and transport of water through these cells is expedited by the removal of cytoplasm during cell differentiation and maturation, their evolution required the capacity for controlled cell death (i.e., apoptosis). Lignification rigidified cell walls in two ways. It provided a bulking agent that increases cell wall stiffness and hydrophobicity. Although not found in bryophytes (e.g., Scheirer, 1973; Hébant, 1977; Ye and Zhong, 2022), lignified secondary cell walls likely evolved as a mechanism to cope with increasing plant size and to resist the implosive collapse of water-conducting cells resulting from the high tensile stresses in water columns due to excessive evapotranspiration. Such lignified walls are largely impervious to the passage of water molecules, necessitating the presence of cell wall perforations (pits) permitting the passage of water molecules.

Our conceptualization of the evolution of the first vascular plants has relied heavily on the appearance of xylem in the fossil record as it is the “hard part” of land plants (e.g., Edwards, 2003; Taylor et al. 2009). However, it must not escape attention that xylem is only one of two vascular tissue systems. Therefore, it would be incorrect to taxonomically exclude an organism possessing phloem but lacking xylem from being a vascular plant. This caveat is important because it is possible that the evolution of phloem predated the evolution of xylem. Phloem can conduct large quantities of water as well as dissolved metabolites and nutrients. It is also potentially energetically advantageous to lose the capacity to form xylem, provided phloem is able to transport nutrients and water in sufficient quantities. In the absence of high evapotranspiration rates, a phloem-like tissue system (functionally analogous to moss leptoids) can provide the bulk transport of water as well as metabolites and nutrients in comparatively short plants. Such living transport cells would not involve the acquisition of the developmental controls allowing apoptosis, required by xylem. The fossil record makes testing this hypothesis difficult because phloem is a delicate tissue and typically evades preservation much more than xylem cell-types. Nevertheless, an illustrative example is provided by the Devonian plants called *Rhynia major* and *R. gwynne-vaughanii. Rhynia major* was originally described as possessing xylem and phloem (Kidston and Lang, 1920). However, a re-examination demonstrated that it lacked xylem, resulting in its removal from the genus *Rhynia* and assignation to the new genus *Aglaophyton* (Edwards, 1986). Whether this absence of xylem reflects the pleiotropic loss of the ability to produce xylem or the appearance of phloem before xylem, describing *Aglaophyton* as a pre-vascular plant would be unwarranted if it possessed phloem. A similar argument can be made in the case of *Horneophyton*, which is purported to lack conventional tracheids (Kenrick and Crane, 1997).

## Branched cylindrical architectures

**Fig. 6:**
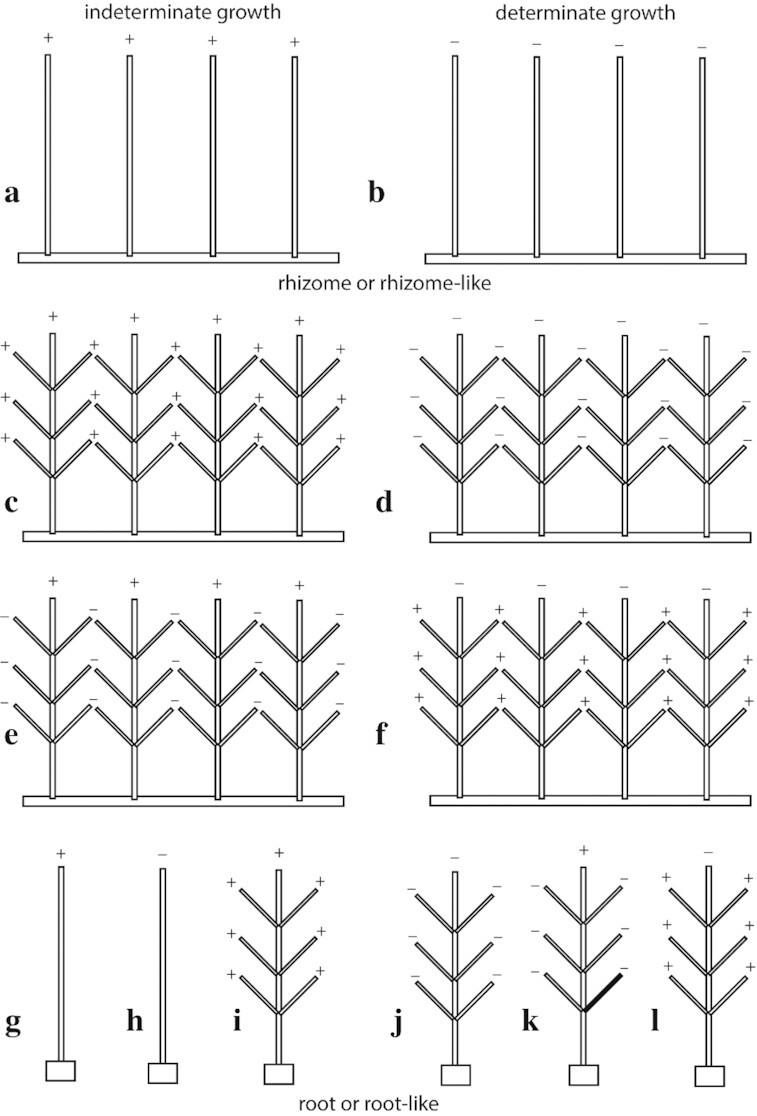
Schematics of a highly redacted morphospace depicting twelve hypothetical land plant architectures based on the permutations of only three variables: (1) the presence or absence of rhizomatous (or rhizomatous-like, e.g., moss protonema) or central root system (or root-like, e.g., liverwort gametophyte foot) anchorage, (2) the presence or absence of apical meristematic indeterminate or determinate growth (denoted by + and −, respectively), and (3) the presence or absence of lateral branching (the former shown as opposite branching for convenience, although alternative patterns or branching such as whorled, spiraled, or random are permitted). Simple dichotomous and overtopped branching patterns are subsumed in Figs. 6c–d. The presence of reproductive organs or of leaves or leaf-like structures (e.g., moss phyllids) is not depicted but is not excluded as a possibility for any of the 12 architectures. Although all 12 permutations are theoretically possible, eight have terminal or lateral branches that are mechanically unstable unless axes have the capacity to produce secondary growth producing wood or wood-like tissues such as sclerenchyma (i.e., a, c, e, f, g, i, k, and l). Examples of extant and extinct taxa (known to the authors) are provided when possible in the text (note: the absence of an example does not indicate that the architecture was not achieved during the evolution of plants).

This review of plant body plans concludes by considering branched variants within a simple morphospace based on three variables: (1) whether axes are determinate or indeterminate in growth; (2) whether individual axes branch; and (3) whether the system providing anchorage to vertical axes is rhizomatous or rhizomatous-like, or root or root-like (Fig. 6). These three variables yield 12 architectures grouped into two classes based on their mode of anchorage (i.e., rhizomatous versus a basal root system). Each of the 12 architectures provides a scaffold to which vegetative or reproductive organs can be attached (e.g., the phyllids of mosses, or lycophyte and euphyllophyte leaves, or sporangia or seed-bearing organs, respectively). The morphospace does not consider variants of the geometric arrangement of vertical or horizontal axes (e.g., whether axes are arranged in spirals or whorls). Nor does it consider the plethora of convergent solutions to increasing photosynthetic surface area called “leaves” (Harrison et al. 2005; Harrison and Morris, 2017; Hrabovský, 2020a, 2020b), a morphological innovation posited to have been made possible by changes in atmospheric composition during the Devonian (Osborne et al. 2004). In addition, the morphospace does not consider the presence or absence of secondary growth, which would be required to maintain the mechanical stability of vertical axes with indeterminate growth (e.g., if terrestrial, the architectures shown in Figs. 6a and c cannot be vertically sustained in the absence of secondary growth). Nor does it purport to provide insights into evolutionary changes in ontogeny, many of which are carefully discussed by Stein and Boyer (2006).

Despite its simplicity, the morphospace identifies branching patterns that mimic those observed among extinct and extant terrestrial plants as well as bryophytes and algae. For example, arguably the simplest architectures consist of a rhizome-like anchorage system bearing indeterminate or determinate unbranched or dichotomously vertical axes supported by rhizomes (Figs. 6a–d) that mimic the growth patterns of some rhyniophytes and lycophytes (e.g., the fossil *Cooksonia* and the lycophyte *Huperzia*) as well as the gametophytes of mosses (Fig. 6c) and some algae (e.g., extant species of *Caulerpa* and the extinct alga *Paleoporella*). Likewise, Figs. 6d–e are similar to the branching patterns of the extinct Aneurophytales, some *Equisteum* species, the extinct sphenopsid *Calamities*, as well as species of grasses and many other herbaceous rhizomatous angiosperms (Bell and Tomlinson, 1980).

Turning to the architectures anchored by roots or root-like systems (Figs. 6g–l), Fig. 6g redacts the growth patterns of many palms, cycads, and even that of the brown alga *Macrocystis*. Fig. 6h reflects single-stemmed determinate plants, including other palms (e.g., *Corypha*), many herbaceous angiosperms (e.g., *Helianthus*, the sunflower), and potentially some members of the extinct cycad-like Bennettiales. This determinate growth model also occurs in some branched angiosperms (Fig. 6j), as for example in *Tachigalia versicolor* in the Fabaceae and possibly other species in this genus (Foster, 1977), as well as the geometry of some dasycladalian algae (e.g., *Triploporella remesii)*. Finally, the vast majority of angiosperm and gymnosperm trees and shrubs conform with Fig. 6i.

Nevertheless, as in the case of many other morphospaces (see Raup, 1961; Raup and Michaelson, 1965; Mitteroecker and Hutteger, 2009; Gerber, 2017), not all of the architectures depicted in Fig. 6 are mechanically feasible. For example, we find it difficult to identify any example of the architectures shown in Figs. 6f, k, or l. The morphospace is also bedeviled by the fact that plants exhibiting outwardly similar morphologies might have arrived at the same architecture via very different developmental pathways. Further, it is also possible that some architectures could “convert” into another. For example, the architectures depicted in Figs. 6a, c, e, g, i, and k all possess indeterminate axial growth that can ultimately exceed the ability of its anchoring system to support its mass against gravity, leading to either an enforced scrambling growth habit in the absence of secondary growth or even mechanical collapse despite the production of secondary tissues. By example, architecture Fig. 6e is mechanically unstable, and, aided by adventitious rooting, indeterminate apical growth could create a horizontal rhizome, while the branches create reiterated determinate apical axes, generating the morphology depicted in Fig. 6b. Similarly, in the absence of secondary growth, Fig. 6c could mechanically fail and generate Fig. 6a.

Clearly, a greater complexity of form than depicted in Fig. 6 can be envisioned based upon growth dynamics, the modes of branching, axis differentiation, and the placement of leaves and reproductive organs (e.g., Stein and Boyer, 2006). Nor does the morphospace consider construction costs or the optimization of transport distances (e.g., Conn et al. 2017). Although these considerations generate a broader range of hypothetical architectures, to date only 23 architectural models have been recognized as represented by living and extinct plants (Hallé et al. 1978; Barthélémy and Caraglio, 2007), a diversity of which appears with the earliest appearance of arborescence in the Devonian (e.g., Hallé et al. 1978, p. 263–8). Importantly, the architectural affinities of their herbaceous precursors in the earlier Devonian and Silurian floras have not been explored. Nor has the potential for the application of this approach to identify models for different rooting structures been explored. Although many roots arise endogenously, others exhibit features observed in aerial axes, such as overtopping, which can exhibit complex behavior.

## Summary

A transition from a simple unicellular body plan into a multicellular body plan that has undergone subsequent evolutionary elaboration has been observed (or at least postulated) to occur many times in the history of eukaryotes. A broad review of the fossil record of the land plants likewise reveals a history of axiation and its subsequent elaboration, facilitated by the elaboration of meristematic activity and the exploitation of ancient biosynthetic pathways and organographic toolkits as revealed by molecular studies of representative extant green algae. The co-option of ancestral traits and their subsequent deployment to achieve new adaptive solutions, as illustrated by the embellishment of the shikimate pathway and the evolution of increasingly elaborate branching architectures, is a theme repeatedly seen across all major eukaryotic photosynthetic and non-photosynthetic clades. However, we suggest that this theme is nowhere better illustrated than in the Viridiplantae, arguably by virtue of their morphological and anatomical simplicity.

## Data Availability

No data-sets were generated for the purpose of writing this paper or preparing figures.
